# The complete chloroplast genome of *Aesculus chinensis* var. *wilsonii*

**DOI:** 10.1080/23802359.2020.1780972

**Published:** 2020-06-25

**Authors:** Zhige Liu, Jingjing Zhang, Yuxin Zhou, Yifei Liu, Zhigang Hu, Guohua Zheng, Zhaohua Shi

**Affiliations:** a Pharmacy Faculty, Hubei University of Chinese Medicine, Wuhan, China; b Wuhan Aimin Pharmaceutical Co., Ltd. R&D Center, Ezhou, Hubei, China

**Keywords:** *Aesculus chinensis* var. *wilsonii*, chloroplast genome, phylogeny

## Abstract

In this study, we sequenced the complete chloroplast (cp) genome of *Aesculus chinensis* Bunge var. *wilsonii* (Rehder) Turland & N. H. Xia and compared it with cp genomes of congeneric species. The cp genome of *A. chinensis* var. *wilsonii* is a circular molecule, 156,211 bp in length, with typical quadripartite structure. It has one large single copy (LSC) region of 85,211 bp and one small single copy (SSC) region of 18,124 bp that are separated by two inverted repeat regions (IR) of 26,438 bp. The cp genome encodes 133 genes comprising 85 protein-coding genes, 40 tRNA genes, and eight rRNA ribosomal genes. The overall GC content of the cp genome of *A. chinensis* var. *wilsonii* is 37.93%. We conducted amaximum likelihood phylogenetic analysis, which revealed that *A. chinensis* var. *wilsonii* is sister to *A. wangii* and has a close relationship with *Acer* L. (maples). We expect that the cp genome of *A. chinensis* var. *wilsonii* will be useful for DNA barcoding and species delimitation for this species as well as future studies on the conservation, taxonomy, and evolutionary relationships of *Aesculus* L.

*Aesculus chinensis* Bunge var. *wilsonii* (Rehder) Turland & N. H. Xia (Hippocastanaceae, Sapindales) is a deciduous tree that is primarily distributed within the Chinese provinces of Hubei, Sichuan, and Guizhou (Xiu et al. 2002) and is important in traditional Chinese medicine. Medicinal properties of the plant derive from its seeds, which contain the bioactive compound, aescin. The seeds of *A. chinensis* var. *wilsonii* exhibit a wide range of pharmacological activities, such as reducing liver toxins, alleviating stress, and reducing pain, and aescins are presently used clinically to treat general malaise of the lungs and stomach and pain associated with abdominal and breast swelling (Liu and Zhou 2010; Sirtori 2001).

Prior studies on *A. chinensis* var. *wilsonii* have focused primarily on its bioactive compounds and pharmacological uses (Chen et al. 2000). There have been few reports on development of molecular genetic markers for barcoding and species delimitation of *A. chinensis* var. *wilsonii*, despite this being critical for detecting purity for clinical applications as well as having implication for taxonomic, conservation, ecological, and evolutionary research. Broadly, there has been a lack of development of genetic resources for *Aesculus* L., although some progress has been made for other species within Sapindales (Bachelier and Endress 2009; Gadek et al. 1996). Molecular genetic resources supporting species identification and other research priorities for *A. chinensis* var. *wilsonii* and the genus *Aesculus* are needed.

We collected dry leaves from one individual of *A. chinensis* var. *wilsonii* in Zhuxi of Hubei province (109°43′E, 31°53′N, 1160 m). Voucher specimens were deposited in Hubei University of Chinese Medicine Herbarium (herbarium number: HB3641MT12). We used the leaves to extract total genomic DNA with a modified cetyltrimethylammonium bromide (CTAB) method (DOYLE 1987). We carried out sequencing on an Illumina HiSeq2000 high-throughput sequencer, which generated approximately 5.66 Gb raw data of 150 bp paired-end reads. We filtered the raw reads in SOAPnuke (Bankevich et al. 2012) to obtain highquality reads. Thereafter, we used SPAdes (Bankevich et al. 2012) to assemble the genome, and primarily used DOGMA (Wyman et al. 2004) and CPGAVAS (Liu et al. 2012) to annotate the cp genome. Finally, the verified complete chloroplast genome sequence of *A. chinensis* var. *wilsonii* was submitted to GenBank (Accession number MT374741). We obtained a total of 20 additional genome sequences of Sapindales from NCBI and aligned them using ClustalW2 (Larkin et al. 2007). We inferred phylogenetic relationships among these representatives of Sapindales using the maximum-likelihood method implemented in RAxML v8.2.12 (Alexandros 2014), all the node reliabilities were computed using 1000 boot strap replicates.

The complete cp genome of *A. chinensis* var. *wilsonii* is 156,211 bp in length. It contains LSC and SCC regions of 85,211 bp and 18,124 bp, respectively, and these are separated by a pair of IR regions that have 26,438 bp in total. The total GC content of the cp genome of *A. chinensis* var. *wilsonii*is 37.93% and is, therefore, similar to *Aesculus wangii* (MF583747) (Zheng et al. 2018), which has 38% GC content. The GC content of the IR regions of the cp genome *A. chinensis* var. *wilsonii* is 42.85% and is higher than that in the LSC and SSC regions, which have 36.15% and 31.92%, respectively. The cp genome of *A. chinensis* var. *wilsonii* encodes 133 genes, including 85 protein-coding genes (PCGs), 40 tRNA genes, and eight rRNA genes. The LSC region contains 58 PCGs and 25 tRNAs. The SSC region contains 11 PCGs and one tRNA. Eight PCGs (*rpl2, rpl23, ycf2, ndhB, rps7, ycf1, rps12 and ycf15*), all rRNAs (*rrn16, rrn23, rrn4.5, and rrn5*) and seven tRNAs (*trnI-CAU, trnL-CAA, trnV-GAC, trnI-GAU, trnA-UGC, trnR-ACG, and trnN-GUU*) are duplicated within the IR regions. Of all the 133 genes, 11 genes (*atpF, rpoC1, ycf3, rpl2, ndhB, ndhA, trnK-UUU, trnL-UAA, trnV-UAC, trnI-GAU, trnA-UGC*) have only one intron and three genes (*clpP, rps12 and ycf3*) have two introns.

The result of the phylogenetic tree showed that *A. chinensis* var. *wilsonii* belongs to the Hippocastanaceaefamily within the order Sapindales. Here, the phylogenetic tree that we reconstructed using maximum likelihood (ML) based on the cp genomes of 20 species from Sapindalesrepresenting five families (Icacinaceae, Sapindaceae, Aceraceae, Hippocastanaceae, and Celastraceae)showed that *A. chinensis* var. *wilsonii* is sister to *A. wangii* (Figure 1). The ML tree also showed that Hippocastanaceaeis sister to Aceraceae with 100% mbs as is consistent.

**Figure 1.**
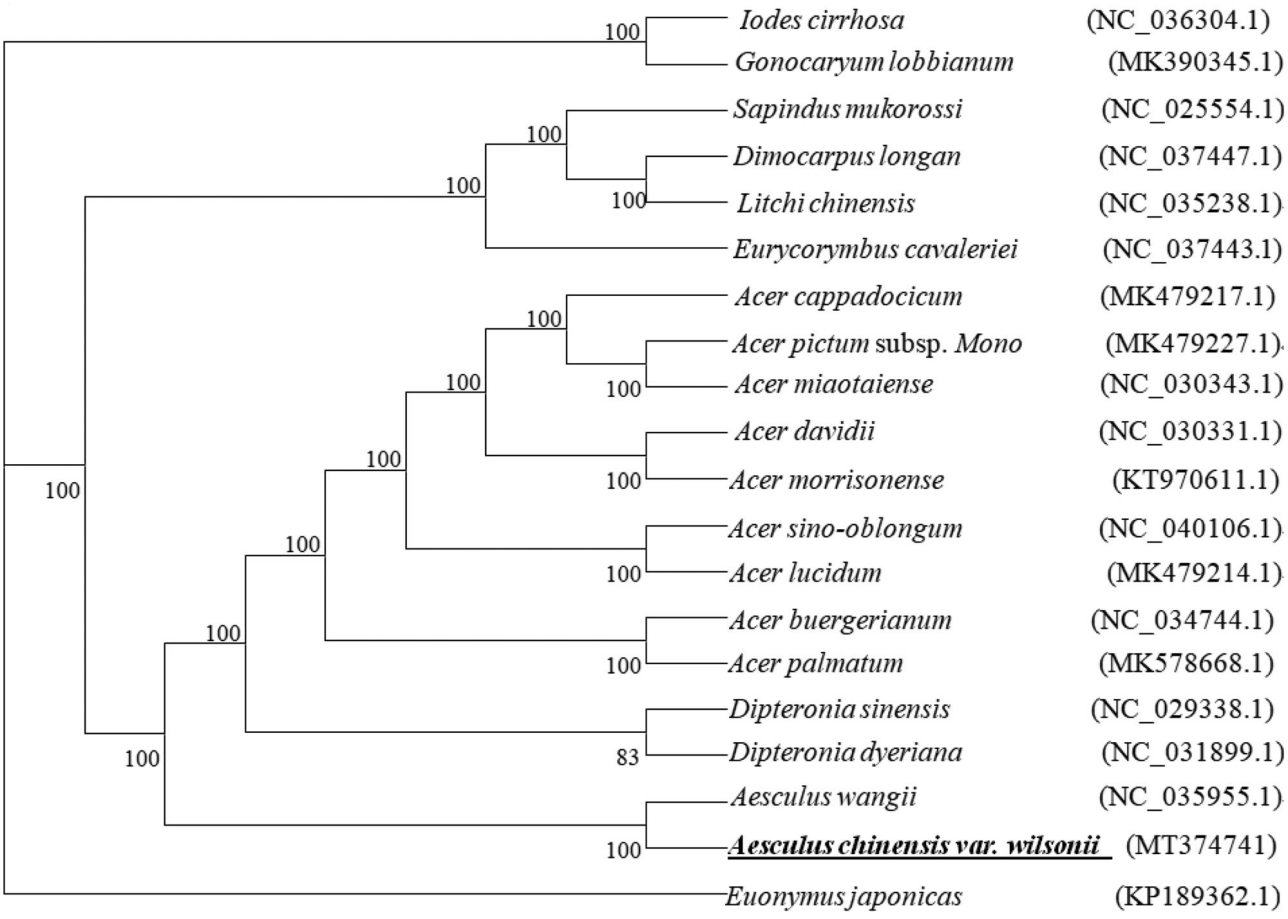
Unrooted maximum likelihood phylogeny of 20 species within Sapindales based on analysis of complete cp genomes. The position of *A. chinensis* var. *wilsonii* is shown in bold and underlined and maximum likelihood bootstrap support is shown above branches.

Here, we provide the first detailed report and analysis of the cp genome of *A. chinensis* var. *wilsonii*. The cp genome exhibited a quadripartite structure typical of vascular plants with one LSC, one SSC, and two IR regions. The cp genomes of *A. chinensis* var. *wilsonii* was highly similar to that of *A. wangii* in terms of the gene content, gene order, and cp genome structure. Phylogenetic relationships among 20 species of Sapindales strongly supported a sister relationship between *A. chinensis* var. *wilsonii* and *A. wangii*. We expect that this draft cp genome of *A. chinensis* var. *wilsonii* will represent a foundational genomic resource for the species.

## Data Availability

The data that support the findings of this study are openly available in NCBI (National Center for Biotechnology Information) (https://www.ncbi.nlm.nih.gov/), reference number: MT374741. This is an Open Access article distributed under the terms of the Creative Commons Attribution License (http://creativecommons.org/licenses/by/4.0/), which permits unrestricted use, distribution, and reproduction in any medium, provided the original work is properly cited.
